# Comparative analysis of amino acid sequences from mesophiles and thermophiles in respective of carbon–nitrogen hydrolase family

**DOI:** 10.1007/s13205-012-0111-3

**Published:** 2013-01-16

**Authors:** Sarita Devi, Nikhil Sharma, Savitri, Tek Chand Bhalla

**Affiliations:** 1 Bioinformatics Centre (Sub-Distributed Information Centre), Himachal Pradesh University, Shimla, Summer Hill 171005 India; 2 Department of Biotechnology, Himachal Pradesh University, Shimla, Summer Hill 171005 India

**Keywords:** Carbon–nitrogen bonds, Nitrilase/cyanide hydratase family, Nitrilase, Cyanide hydratase, Thermostability, Phylogenetic tree

## Abstract

A comparative study of amino acid sequence and physicochemical properties indicates the affiliation of protein from the nitrilase/cyanide hydratase family. This family contains nitrilases that break carbon–nitrogen bonds and appear to be involved in the reduction of organic nitrogen compounds and ammonia production. They all have distinct substrate specificity and include nitrilase, cyanide hydratases, aliphatic amidases, beta-alanine synthase, and a few other proteins with unknown molecular function. These sequences were analyzed for different physical and chemical properties and to relate these observed differences to the thermostability properties, phylogenetic tree construction and the evolutionary relationship among them. In this work, in silico analysis of amino acid sequences of mesophilic (15) and thermophilic (archaea, 15 and bacteria, 15) proteins has been done. The physiochemical properties of these three groups of nitrilase/cyanide hydratase family also differ in number of amino acids, molecular weight, pI values, positively charged ions, i.e. Arg + Lys, aliphatic index and grand average of hydropathacity (GRAVY). The amino acid Ala (1.37-fold) was found to be higher in mesophilic bacteria as compared to thermophilic bacteria but Lys and Phe were found to be significantly high (1.43 and 1.39-fold, respectively) in case of thermophilic bacteria. The amino acids Ala, Cys, Gln, His and Thr were found to be significantly higher (1.41, 1.6, 1.77, 1.44 and 1.29-fold, respectively) in mesophilic bacteria as compared to thermophilic archaea, where Glu, Leu and Val were found significantly high (1.22, 1.19 and 1.26-fold, respectively).

## Introduction

On the basis of structure and sequence analysis, new family of enzyme, termed as nitrilase/cyanide hydratase, was constructed (Brenner 2002) that includes nitrilase cyanide hydratase and cyanide dihydratase, which also incorporated the less closely related aliphatic amidases (Novo et al. 1995). This family is part of a larger group of related proteins, which have been termed CN-hydrolases (Bork and Koonin 1994) or more recently as the nitrilase superfamily (Pace and Brenner 2001). Plants, animals and fungi perform a wide variety of non-peptide carbon–nitrogen hydrolysis reactions using enzymes of the nitrilase superfamily (Pace and Brenner 2001). These nitrilase and amidase reactions (Ambler et al. 1987; Bork and Koonin 1994; Pace and Brenner 2001) produce auxin, biotin, β-alanine and other natural products, and which result in deamination of protein and amino acid substrates, all involve attack of a cyano or carbonyl carbon by a conserved cysteine (Stevenson et al. 1990; Pace and Brenner 2001). Many bacteria and archaea, particularly those with an ecological relationship to plants and animals harbor members of the nitrilase superfamily and utilize the enzymes for chemically similar nitrile or amide hydrolysis reactions or for condensation of acyl chains to polypeptide amino termini (Pace and Brenner 2001). The nitrilase superfamily consists of thiol enzymes involved in natural product biosynthesis and posttranslational modification in plants, animals, fungi and certain prokaryotes. On the basis of sequence similarity and the presence of additional domains, the superfamily is classified into 13 branches, although the substrate specificity is known for only nine branches (Brenner 2002). Only branch one has nitrilase or cyanide hydratase activity, and eight of the remaining branches have amidase or amide condensation activities (Brenner 2002). Genetic and biochemical analysis of the family members and their associated domains helps in predicting the localization, specificity and cell biology of hundreds of uncharacterized protein (Pace and Brenner 2001).

The proteins show significant similarities at the amino acid and protein structure level but the enzymes show many differences in catalytic capability. Nitrilases, while catalyzing the hydration of nitrile to the corresponding acid, vary widely in substrate specificity. Cyanide dihydratase and cyanide hydratase employ inorganic cyanide as the only efficient substrate but produce acid and amide products, respectively. The similarities of all these enzymes at the amino acid level but the functional differences between them provide a platform for the study of structure/function relationships in this industrially important group of enzymes (O’Reilly and Turner 2003).

Cyanide and nitrile hydrolyzing enzymes have been studied in a wide range of microbial species, plants and animal systems. The enzymatic conversion of inorganic cyanide/nitrile to the corresponding acid can take place by a one-step process as exemplified by nitrilases and cyanide dihydratases or by a two-step process with an amide intermediate as is the case with nitrile hydratases and cyanide hydratases. Cyanide hydratase, although functionally different, shows no relationship to the more functionally similar nitrile hydratase (Wang and VanEtten 1992; Cluness et al. 1993). They have cyanide-hydrating activity but the enzymes differ in the product produced or in substrate specificity. Cyanide dihydratase and cyanide hydratase enzymes show high specificity for inorganic cyanide showing very little activity with nitriles, while nitrilases in general show activity with a broad range of nitrile substrates. Nitrilases and cyanide dihydratase produce mainly an acid product, while cyanide hydratase produces the amide product from inorganic cyanide. The nitrilases are important for their potential application in biotransformation particularly for the production of fine chemicals for the pharmaceutical industry (Kobayashi and Shimizu 2000; Banerjee et al. 2002), while inorganic cyanide-hydrating enzymes have application in the bioremediation of cyanide bearing waste (Dubey and Holmes 1995; O’Reilly and Turner 2003). Nitrilase-related sequences are also found in phylogenetically isolated prokaryotes that appear to have an ecological relationship to plants and animals. The nitrilase superfamily therefore probably emerged prior to the separation of plants, animals and fungi, radiated into families, and then spread laterally to bacteria and archaea. Some branches of the nitrilase superfamily are found only in prokaryotes; members of these branches may constitute rational antibiotic targets (Pace and Brenner 2001).

A number of physiochemical properties, e.g. number of amino acid residues, molecular mass, theoretical pI, amino acid composition, negatively charged residues (Asp + Glu), positively charged residues (Arg + Lys), atomic composition, total number of atoms, extinction coefficients (M^−1^ cm^−1^) at 280 nm, instability index, aliphatic index, grand average hydropathicity (GRAVY), etc. of enzymes greatly influence their applications and need to be carefully studied. These properties can be either determined experimentally or deduced from the in silico analysis of amino acid sequences of enzymes available in the databases. Latter approach seems to be attractive for comparison of large number of proteins/enzymes provided the amino acid sequences are available. In the present study, we report some physiochemical properties of proteins from nitrilase/cyanide hydratase family deduced from the in silico analysis of their amino acid sequences and also constructed the phylogenetic tree for their evolutionary relation.

## Materials and methods

### Data collection and analysis

Information about the affinity for protein from nitrilase/cyanide hydratase family of some microorganisms was obtained from the National Centre for Biotechnology Information (NCBI, http://www.ncbi.nlm.nih.gov/protein) and from the NCBI Bioproject (http://www.ncbi.nlm.nih.gov/bioproject/). Amino acid sequences for all the forty five microorganisms having experimentally proved substrate specificity as well as complete protein sequences which are not fragmented, pseudo, putative or hypothetical (Tables 1, 2, 3). The amino acid sequences of nitrilase/cyanide hydratase family were downloaded from the ExPASy proteomic server. Physiochemical data were generated from the SwissProt and Expert Protein Analysis System (ExPASy) that is the proteomic server of Swiss Institute of Bioinformatics (SIB). FASTA format of sequences were used for analysis.

**Table 1:** Name of mesophilic microorganism with there accession number for nitrilase/cyanide hydratase family with accession number

S. no.	Accession no.	Microorganism	Temperature (°C)	References
1	YP_003995948	*Halanaerobium hydrogeniformans*	32–42	Brown et al. 2011
2	ZP_09101712	*Desulfotomaculum gibsoniae* DSM 7213	37	Kuever et al. 1999
3	ZP_08919207	*Thermobacillus composti* KWC4	50	Watanabe et al. 2007
4	ZP_09085711	*Mesorhizobium amorphae* CCNWGS0123	28	Hao et al. 2012
5	ZP_09968828	*Serratia sp.* M24T3	30	Proença et al. 2012
6	EGD48755	*Clostridium papyrosolvens* DSM 2782	25	Nishiyama et al. 2009
7	YP_004828482	*Kangiella koreensis* DSM 16069	30–37	Yoon et al. 2004
8	ADY60178	*Planctomyces brasiliensis* DSM 5305	30	Fukunaga et al. 2009
9	YP_004092573	*Ethanoligenens harbinense* YUAN-3	20–44	Xing et al. 2006
10	YP_003914730	*Ferrimonas balearica* DSM 9799	28	Rossello-Mora et al. 1995
11	AEG54379	*Sinorhizobium meliloti* AK83	25–30	Galardini et al. 2011
12	YP_004604016	*Flexistipes sinusarabici* DSM 4947	45–50	Fiala et al. 1990
13	ADJ26301	*Dehalogenimonas lykanthroporepellens* BL-DC-9	30	Lucas et al. 2010c
14	ABX36273	*Delftia acidovorans* SPH-1	30	Schleheck et al. 2004
15	YP_003891848	*Sulfurimonas autotrophica* DSM 16294	24	Sikorski et al. 2010

http://www.ncbi.nlm.nih.gov/bioproject/

**Table 2:** Name of thermophilic bacteria with there accession number for nitrilase/cyanide hydratase family with accession number

S. no.	Accession no.	Microorganism	Temp (°C)	References
1	gi56554251	*Bacillus Smithii*	60	Hourai et al. 2003
2	ADQ46841	*Caldicellulosiruptor kronotskyensis* 2002	70–78	Miroshnichenko et al. 2008
3	ACX52587	*Ammonifex degensii* KC4	70	Kerfeld et al. 2009
4	ACR79531	*Kosmotoga olearia* TBF 19.5.1	70	Swithers et al. 2011
5	YP_004340560	*Hippea maritime* DSM 10411	40–75	Huntemann et al. 2011
6	YP_001306296	*Thermosipho melanesiensis* BI429	70	Antoine et al. 1997
7	YP_001244336	*Thermotoga petrophila* RKU-1	80	Takahata et al. 2001
8	AEH48988	*Geobacillus thermoglucosidasius* C56-YS93	55–65	Lucas et al. 2011a
9	YP_002730079	*Persephonella marina* EX-H1	73	Reysenbach et al. 2009
10	ABC20379	*Moorella thermoacetica* ATCC 39073	60	Qingyan et al. 2009
11	ZP_01666349	*Thermosinus carboxydivorans* Nor1	60	Sokolova et al. 2004
12	YP_004516912	*Desulfotomaculum kuznetsovii*	55–60	Anandkumar et al. 2009
13	YP_004437096	*Thermodesulfobium narugense* DSM 14796	28–65	Lucas et al. 2011b
14	EEU01226	*Clostridium thermocellum* DSM 2360	60	Ng et al. 1981
15	YP_003826095	*Thermosediminibacter oceani* DSM 16646	68	Pitluck et al. 2010

http://www.ncbi.nlm.nih.gov/bioproject/

**Table 3:** Name of thermophilic archaea with there accession number for nitrilase/cyanide hydratase family with accession number

S. no.	Accession no.	Microorganism	Temp (°C)	References
1	BAJ46738	*Candidatus Caldiarchaeum subterraneum*	70–80	Nunoura et al. 2011
2	YP_920845	*Thermofilum pendens* Hrk 5	88	Anderson et al.^2008^
3	YP_003400513	*Archaeoglobus profundus* DSM 5631	85	von Jan et al. 2010
4	ADC65733	*Ferroglobus placidus* DSM 10642	85	Lucas et al. 2010a
5	ADD08508	*Aciduliprofundum boonei* T469	70	Lucas et al. 2010b
6	AFH42973	*Fervidicoccus fontis Kam* 940	50–92	Lebedinsky et al. 2012
7	YP_003668629	*Staphylothermus hellenicus* DSM 12710	85	Arab et al. 2000
8	ACB07703	*Candidatus Korarchaeum cryptofilum* OPF8	60–90	Elkins et al. 2008
9	ADN51834	*Thermoproteaceae Vulcanisaeta* DSM 14429	90	Mavromatis et al. 2010
10	ABP51566	*Pyrobaculum arsenaticum* DSM 13514	Hyperthermophilic	Copeland et al. 2007
11	YP_004342152	*Archaeoglobus veneficus* SNP6	75	Lucas et al. 2011a
12	ACX92976	*Sulfolobus solfataricus* 98/2	80	Lucas et al. 2009
13	ACP47189	*Sulfolobus islandicus* Y.G.57.14	75–80	Reno et al. 2009
14	ACP39491	*Sulfolobus islandicus* M.14.25	75–85	Reno et al. 2009
15	ADB87996	*Sulfolobus islandicus* L.D.8.5	75–80	Whitaker et al. 2003

http://www.ncbi.nlm.nih.gov/bioproject/

### Sequence alignment and dendrogram construction

The program Clustal X (Larkin et al. 2007) was used for multiple sequence alignment; Phylip-69 was used for dendrogram construction by neighbor-joining (NJ) method. The dendrogram was edited by Dendroscope (Huson et al. 2007).

### Deduction of physiochemical parameters generation using online tools

Various tools in the proteomic server (ProtParam, Protein calculator, Compute pI/Mw, ProtScale) were applied to calculate/deduce different physiochemical properties of amidases from the protein sequences (Kyte and Doolittle 1982). The molecular weights (kDa) of these sequences were calculated by the addition of average isotopic masses of amino acid in the protein and deducting the average isotopic mass of one water molecule. The pI was calculated using pK values of amino acid (Bjellqvist et al. 1993). The atomic composition of these sequences was derived using the ProtParam tool, available at ExPASy. The extinction coefficient of various proteins from nitrilase/cyanide hydratase family was calculated using the following equation (Stanley et al. 1989): 

The values of aliphatic index of various sequences were obtained using ProtParam (ExPASy) tool (Kyte and Doolittle 1982). The instability index and grand average of hydropathicity (GRAVY) were estimated following the method of Guruprasad et al. (1990) and Kyte and Doolittle (1982), respectively. The number of amino acids was calculated with the help of ProtParam tool of the exposure proteomic server submitted as raw sequence in the fasta format.

### Statistical analysis

Various parameters were calculated using statistical package ‘Assistat version-7.6 beta 2012’ for the *p* value regarding the same. An analysis of variance (ANOVA) or one way ANOVA follows the rule of null hypothesis which implies the data to be homogenous. *F* test was used to determine the statistical significance. When significant effects were detected, a Tukey test was applied for all pair-wise comparisons of mean responses. *F* test helps to calculate the means to the variance within the samples, whereas *T* test or the Tukey test covers at least two groups taken with equal set or the homogenous set of data with equal number of samples.

## Results and discussion

### Phylogenetic tree construction

**Fig. 1:**
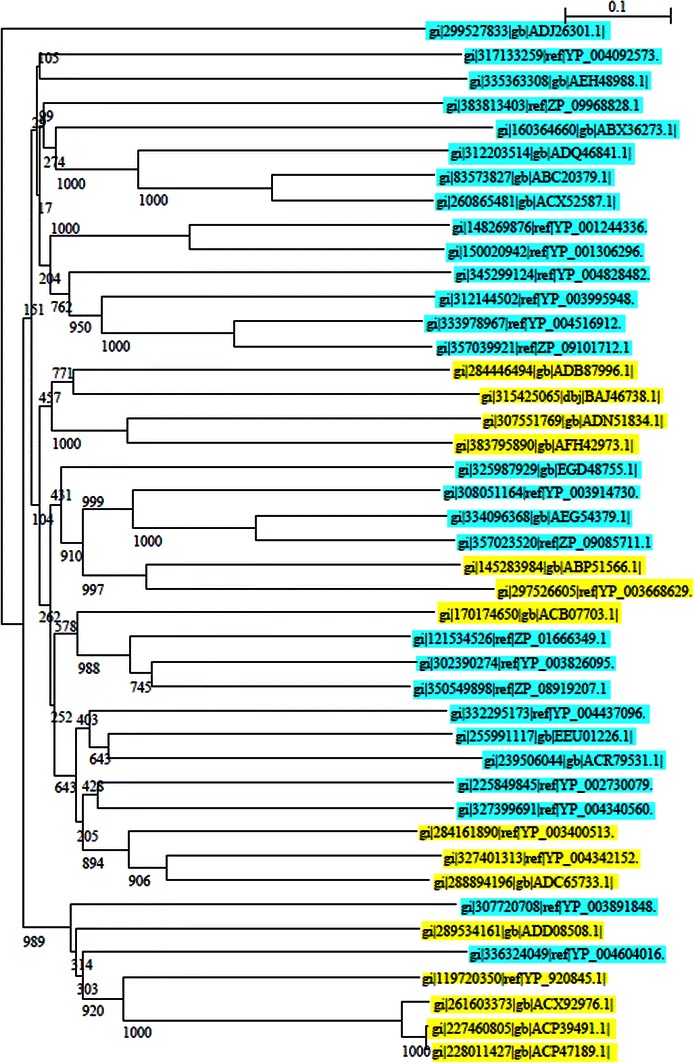
Phylogenetic tree of protein sequences of nitrilase/cyanide hydratase family from bacterial (Fermicutes, proteobacteria and bacteria) and archaeal source organism constructed by NJ method, where bacterial species are shown in*light blue* and archaeal sp. is shown as *yellow* in color. Numbers at nodes are bootstrap support percentages from PhyML (1000 replicates). The *scale bar* indicates the average number of amino acid substitutions per site

In the present study to visualize the evolutionary relationship between the bacterial and archaeal sources from the protein sequences belonging to nitrilase/cyanide hydratase family, a total of forty three protein sequences of nitrilase/cyanide hydratase family from bacterial and archaeal source organisms were subjected to phylogenetic tree construction revealed three major clusters (Fig. 1). One has only bacteria, another have both bacterial and archaeal species and one with dominance in archaeal strains. However, in second cluster nearly about all bacterial and archaeal species were found together in a corner which indicates the functional as well as structural similarity among these genera according to protein sequence. As far as phylogenetic tree was concerned, the bacterial species of second cluster have some structural similarity with those archaeal species but that showed very distinct relationship with respect to bootstrap values. The archaea are presently recognized as one of the two main domains of prokaryotes (Woese et al. 1990; Woese 1987). The majority of genes that indicate archaea to be different from eubacteria are for information transfer processes such as DNA replication, transcription and translation (Olsen et al. 1994; Rivera et al. 1999), and these processes are of fundamental importance. It has been assumed that these differences arose in the universal ancestor before the separation of these two domains. Woese (1998) and Kandler (1998) have suggested that these two domains as well as the eukaryotic cells evolved from a pre-cellular community containing different types of genes by a process that led to fixation of specific subsets of genes in the ancestors of these domains. These pre-cellular entities are postulated to have no stable genealogy or chromosome and also lacking a typical cell membrane, thus allowing unrestricted lateral gene transfers (Woese 1998; Kandler 1998). According to these proposals, all differences between archaea and bacteria originated at a pre-cellular stage by non-Darwinian means, but they suggest no rationale as to how or why the observed differences between these two groups arose or evolved. Cavalier-Smith (2002) has suggested the possibility of archaea evolving from Gram-positive bacteria as an adaptation to hyperthermophile or hyperacidity, but it does not explain how various differences in the information transfer genes which distinguish archaea from bacteria arose.

### Physiochemical parameter analysis

After finding the evolutionary relationships among these sequences, the attempts to find differences between the physiochemical properties of forty five amino acid sequences of mesophilic bacteria (15), thermophilic bacteria (15) and thermophilic archaea (15) from nitrilase/cyanide hydratase family have been done (Tables 4, 5, 6). The comparison of mesophilic and thermophilic bacteria for the sequences from nitrilase/cyanide hydratase family has been done and the total number of amino acid residues, molecular weight, theoretical pI and negatively charged residues (Asp + Gln) in these sequences differed substantially as mesophilic bacteria have more number of amino acid residues ranging between 240 and 345 amino acids whereas thermophilic bacteria ranging between 229 and 291. The molecular weight and negatively charged residues (Asp + Gln) in nitrilase/cyanide hydratase sequences of mesophilic bacteria were found to be insignificantly high as compared to the thermophilic bacteria (1.08, 1.12-fold, respectively). Theoretical pI varied between 5.06 and 8.75 in case of nitrilase/cyanide hydratase of mesophilic bacteria and it was found to be 5.44 and 9.68 for nitrilase/cyanide hydratase sequences of thermophilic bacteria. It was further found that the average pI value of thermophilic bacteria was significantly higher than that of mesophilic bacterial nitrilases/cyanide hydratases (1.2-fold). The aliphatic index has the significant effect (1.12-fold) by comparing the physicochemical parameters of nitrilase/cyanide hydratase sequences of thermophilic bacteria and archaea. Various factors have been shown to contribute to the stability of proteins from thermophiles (Russell et al. 1997; Jaenicke and Bohm 1998; Ladenstein and Antranikian 1998). The importance of electrostatic interactions (Goldman 1995; Hennig et al. 1995; Xiao and Honig 1999), increased compactness, shortening of loops, increased hydrophobicity and decreased flexibility of α-helical segments and subunit interfaces (Kelly et al. 1993; Russell et al. 1997) have been proposed as important factors conferring thermal stability. All these studies suggest that in thermophilic proteins, stability is achieved through cooperative optimization of several subtle factors rather than any one predominant interaction.

**Table 4:** Comparative analysis of physiochemical properties of mesophilic and thermophilic bacteria for nitrilase/cyanide hydratase family

Parameters	SS	Microorganisms															(*P* < 0.01)
		1	2	3	4	5	6	7	8	9	10	11	12	13	14	15	
No. of amino acids	MB	267	277	270	290	302	267	273	346	301	274	285	276	257	345	240	10.26**
	TB	229	231	242	244	261	264	267	291	246	245	258	280	255	257	268	
Molecular weight (Da)	MB	30,207.9	30,481.8	30,154.3	30,989.0	33,743.5	29,661.0	30,561.7	38,175.3	33,551.0	30,114.2	30,536.5	31,413.9	28,169.9	38,564.7	27,263.3	6.88*
	TB	26,663.2	26,290.3	26,544.7	28,797.8	29,967.6	30,380.3	30,054.8	32,751.2	28,060.4	26,259.1	28,603.9	31,420.9	29,549.0	29,480.8	30,495.3	
Theoretical pI	MB	8.51	8.75	5.39	5.44	5.39	5.66	5.39	5.06	5.58	5.92	5.70	5.71	5.06	5.69	5.21	6.43*
	TB	5.44	5.80	8.32	6.25	8.11	9.68	8.30	5.69	8.82	5.98	6.25	6.19	8.60	5.86	6.87	
Negatively charged residue (Asp + Glu)	MB	34	29	43	39	39	32	36	46	37	31	37	36	36	44	33	4.60*
	TB	39	31	28	36	31	28	32	37	32	26	32	35	32	41	38	
Positively charged residue (Arg + Lys)	MB	37	33	34	31	29	28	26	33	31	25	31	32	25	35	25	2.23 ns
	TB	30	27	30	34	33	41	34	29	36	23	30	32	36	37	38	
Extinction coffiecient (M^−1^ cm^−1^) at 280 nm	MB	29,340	25,900	30,940	25,440	33,350	22,920	36,900	42,400	64,290	43,430	23,950	33,350	51,450	54,320	32,430	1.59 ns
	TB	46,870	24,870	15,930	42,860	40,910	20,860	23,380	34,380	27,390	18,910	40,910	22,920	22,920	38,390	42,400	
Instability index	MB	38.55	42.24	44.66	27.72	39.62	25.92	39.53	50.40	33.70	37.30	24.07	43.34	47.65	38.09	41.79	2.77 ns
	TB	31.36	36.78	38.75	42.75	25.02	30.71	31.77	25.23	40.22	26.40	35.86	35.64	32.46	45.16	32.86	
Aliphatic index	MB	102.36	86.64	90.04	93.41	89.77	89.48	90.70	87.23	74.25	91.61	84.00	86.20	84.36	69.54	101.50	0.79 ns
	TB	74.41	95.50	100.91	68.81	92.64	100.83	95.24	85.05	96.67	95.71	95.31	89.82	88.63	84.98	100.34	
Grand average of hydropathicity (Gravy)	MB	−0.142	−0.199	−0.278	0.072	−0.180	−0.086	−0.210	−0.206	−0.370	−0.216	−0.094	−0.132	−0.135	−0.397	−0.015	0.23 ns
	TB	−0.561	−0.144	0.002	−0.436	−0.196	−0.071	0.030	−0.192	−0.315	0.098	0.001	−0.235	−0.230	−0.422	−0.169	

Substarte specificity: *MB* Mesophilic bacteria, (1) *Halanaerobium hydrogeniformans* (2) *Desulfotomaculum gibsoniae* DSM 7213 (3) *Thermobacillus composti* KWC4 (4) *Mesorhizobium amorphae* CCNWGS0123 (5) *Serratia sp.* M24T3 (6) *Clostridium papyrosolvens* DSM 2782 (7) *Kangiella koreensis* DSM 16069 (8) *Planctomyces brasiliensis* DSM 5305 (9) *Ethanoligenens harbinense* YUAN-3 (10) *Ferrimonas balearica* DSM 9799 (11) *Sinorhizobium meliloti* AK83 (12) *Flexistipes sinusarabici* DSM 4947 (13) *Dehalogenimonas lykanthroporepellens* BL-DC-9 (14) *Delftia acidovorans* SPH-1 (15) *Sulfurimonas autotrophica* DSM 16294

*TB* Thermophilic bacteria (1) *Bacillus Smithii* (2) *Caldicellulosiruptor kronotskyensis* 2002 (3) *Ammonifex degensii* KC4 (4) *Kosmotoga olearia* TBF 19.5.1 (5) *Hippea maritima* DSM 10411 (6) *Thermosipho melanesiensis* BI429 (7) *Thermotoga petrophila* RKU-1 (8) *Geobacillus thermoglucosidasius* C56-YS93 (9) *Persephonella marina* EX-H1 (10) *Moorella thermoacetica* ATCC 39073 (11) Thermosinus carboxydivorans Nor1 (12) *Desulfotomaculum kuznetsovii* (13) *Thermodesulfobium narugense* DSM 14796 (14) *Clostridium thermocellum* DSM 2360 (15) *Thermosediminibacter oceani* DSM 16646

** Significant at a level of 1 % of probability (*P* < 0.01)

* Significant at a level of 5 % of probability (0.01 ≤ *P* < 0.05)

*ns* non-significant (*P* ≥ 0.05)

**Table 5:** Comparative analysis of physiochemical properties of thermophilic bacteria and archaea for nitrilase/cyanide hydratase family

Parameters	SS	Microorganisms															(*P* < 0.01)
		1	2	3	4	5	6	7	8	9	10	11	12	13	14	15	
No. of amino acids	TA	238	270	279	231	250	255	270	273	265	264	258	249	270	270	270	0.55 ns
	TB	229	231	242	244	261	264	267	291	246	245	258	280	255	257	268	
Molecular weight (Da)	TA	26,838.5	30,000.0	31,149	26,250.9	28,243.9	29,384.0	30,684.6	31,878.7	39,521.7	29,233.4	28,171.4	27,765.2	31,320.0	31,349.1	31,301.0	0.574 ns
	TB	26,663.2	26,290.3	26,544.7	28,797.8	29,967.6	30,380.3	30,054.8	32,751.2	28,060.4	26,259.1	28,603.9	31,420.9	29,549.0	29,480.8	30,495.3	
Theoretical pI	TA	6.04	6.37	5.26	9.39	5.38	5.87	8.42	7.78	5.29	5.54	6.47	5.81	6.04	5.86	5.70	2.32 ns
	TB	5.44	5.80	8.32	6.25	8.11	9.68	8.30	5.69	8.82	5.98	6.25	6.19	8.60	5.86	6.87	
Negatively charged residue (Asp + Glu)	TA	33	31	46	27	37	35	35	37	41	29	33	35	40	41	42	2.80 ns
	TB	39	31	28	36	31	28	32	37	32	26	32	35	32	41	38	
Positively charged residue (Arg + Lys)	TA	32	28	35	37	32	33	37	38	33	26	32	32	37	37	37	0.36 ns
	TB	30	27	30	34	33	41	34	29	36	23	30	32	36	37	38	
Extinction coffiecient (M^−1^ cm^−1^) at 280 nm	TA	24,410	33,585	38,390	15,930	17,420	27,390	47,900	43,320	15,930	39,880	29,910	21,430	44,350	44,350	42,860	0.058 ns
	TB	46,870	24,870	15,930	42,860	40,910	20,860	23,380	34,380	27,390	18,910	40,910	22,920	22,920	38,390	42,400	
Instability index	TA	31.41	38.25	44.54	27.58	40.68	30.18	37.25	51.79	41.18	28.79	33.58	46.11	38.24	36.75	36.82	2.21 ns
	TB	31.36	36.78	38.75	42.75	25.02	30.71	31.77	25.23	40.22	26.40	35.86	35.64	32.46	45.16	32.86	
Aliphatic index	TA	120.29	101.50	98.17	106.28	110.28	95.22	109.67	96.74	90.15	107.39	94.15	102.97	97.48	96.41	96.41	11.01**
	TB	74.41	95.50	100.91	68.81	92.64	100.83	95.24	85.05	96.67	95.71	95.31	89.82	88.63	84.98	100.34	
Grand average of hydropathicity (Gravy)	TA	0.112	−0.086	−0.102	−0.084	0.019	−0.042	−0.094	−0.238	−0.168	−0.016	−0.094	0.010	−0.211	−0.204	−0.212	3.94 ns
	TB	−0.561	−0.144	0.002	−0.436	−0.196	−0.071	0.030	−0.192	−0.315	0.098	0.001	−0.235	−0.230	−0.422	−0.169	

Substrate specificity: *TA* Thermophilic archaea, (1) *Sulfolobus islandicus* L.D.8.5 (2) *Candidatus Caldiarchaeum subterraneum* (3) *Thermofilum pendens Hrk* 5 (4) *Archaeoglobus profundus* DSM 5631 (5) *Ferroglobus placidus* DSM 10642 (6) *Aciduliprofundum boonei* T469 (7) *Fervidicoccus fontis Kam* 940 (8) *Staphylothermus hellenicus* DSM 12710 (9) *Candidatus Korarchaeum cryptofilum* OPF8 (10) *Thermoproteaceae Vulcanisaeta* (11) *Pyrobaculum arsenaticum* DSM 13514 (12) *Archaeoglobus veneficus* SNP6 (13) *Sulfolobus solfataricus* 98/2 (14) *Sulfolobus islandicus* Y.G.57.14 (15) *Sulfolobus islandicus* M.14.25

*TB* Thermophilic bacteria (1) *Bacillus Smithii* (2) *Caldicellulosiruptor kronotskyensis* 2002 (3) *Ammonifex degensii* KC4 (4) *Kosmotoga olearia* TBF 19.5.1 (5) *Hippea maritima* DSM 10411 (6) *Thermosipho melanesiensis* BI429 (7) *Thermotoga petrophila* RKU-1 (8) *Geobacillus thermoglucosidasius* C56-YS93 (9) *Persephonella marina* EX-H1 (10) *Moorella thermoacetica* ATCC 39073 (11) *Thermosinus carboxydivorans* Nor1 (12) *Desulfotomaculum kuznetsovii* (13) *Thermodesulfobium narugense* DSM 14796 (14) *Clostridium thermocellum* DSM 2360 (15) *Thermosediminibacter oceani* DSM 16646

** Significant at a level of 1 % of probability (*P* < 0.01)

* Significant at a level of 5 % of probability (0.01 ≤ *P* < 0.05)

*ns* non-significant (*P* ≥ 0.05)

**Table 6:** Comparative analysis of physiochemical properties of mesophilic bacteria and thermophilic archaea for nitrilase/cyanide hydratase family

Parameters		Microorganisms															(*P* < 0.01)
		1	2	3	4	5	6	7	8	9	10	11	12	13	14	15	
No. of amino acids	MB	267	277	270	290	302	267	273	346	301	274	285	276	257	345	240	8.18**
	TA	238	270	279	231	250	255	270	273	265	264	258	249	270	270	270	
Molecular weight (Da)	MB	30,207.9	30,481.8	30,154.3	30,989.0	33,743.5	29,661.0	30,561.7	38,175.3	33,551.0	30,114.2	30,536.5	31,413.9	28,169.9	38,564.7	27,263.3	4.60*
	TA	26,838.5	30,000.6	31,149.3	26,250.9	28,243.9	29,384.0	30,684.6	31,878.7	29,521.7	29,233.4	28,171.4	27,765.2	31,320.0	31,349.1	31,301.0	
Theoretical pI	MB	8.51	8.75	5.39	5.44	5.39	5.66	5.39	5.06	5.58	5.92	5.70	5.71	5.06	5.69	5.21	0.80 ns
	TA	6.04	6.37	5.26	9.39	5.38	5.87	8.42	7.78	5.29	5.54	5.54	5.81	6.04	5.86	5.70	
Negatively charged residue (Asp + Glu)	MB	34	29	43	39	39	32	36	46	37	31	37	36	36	44	33	0.13 ns
	TA	33	31	46	27	37	35	35	37	41	29	33	35	40	41	42	
Positively charged residue (Arg + Lys)	MB	37	33	34	31	29	28	26	33	31	25	31	32	25	35	25	6.25*
	TA	32	28	35	37	32	33	37	38	33	26	32	32	37	37	37	
Extinction coefficient (M^−1^ cm^−1^) at 280 nm	MB	29,340	25,900	30,940	25,440	33,350	22,920	36,900	42,400	64,290	43,430	23,950	33,350	51,450	54,320	32,430	0.95 ns
	TA	24,410	33,585	38,390	15,930	17,420	27,390	47,900	43,320	15,930	39,880	29,910	21,430	44,350	44,350	42,860	
Instability index	MB	38.55	42.24	44.66	27.72	39.62	25.92	39.53	50.40	33.70	37.30	24.07	43.34	47.65	38.09	41.79	0.08 ns
	TA	31.41	38.25	44.54	27.58	40.68	30.18	37.25	51.79	41.18	28.79	33.58	46.11	38.24	36.75	36.82	
Aliphatic index	MB	102.36	86.64	90.04	93.41	89.77	89.48	90.70	87.23	74.25	91.61	84.00	86.20	84.36	69.54	101.50	20.12**
	TA	120.29	101.48	98.17	106.28	110.28	95.22	109.67	96.74	90.15	107.39	94.15	102.97	97.48	96.41	96.41	
Grand average of hygropathacity (Gravy)	MB	−0.142	−0.199	−0.278	0.072	−0.180	−0.086	−0.210	−0.206	−0.370	−0.216	−0.094	−0.132	−0.135	−0.397	−0.015	4.40*
	TA	0.112	−0.086	−0.102	−0.084	0.019	−0.042	−0.094	−0.238	−0.162	0.076	−0.016	0.010	−0.211	−0.204	−0.212	

Substarte specificity: *MB* Mesophilic Bacteria, (1) *Halanaerobium hydrogeniformans* (2) *Desulfotomaculum gibsoniae* DSM 7213 (3) *Thermobacillus composti* KWC4 (4) *Mesorhizobium amorphae* CCNWGS0123 (5) *Serratia* sp. M24T3 (6) *Clostridium papyrosolvens* DSM 2782 (7) *Kangiella koreensis* DSM 16069 (8) *Planctomyces brasiliensis* DSM 5305 (9) *Ethanoligenens harbinense* YUAN-3 (10) *Ferrimonas balearica* DSM 9799 (11) *Sinorhizobium meliloti* AK83 (12) *Flexistipes sinusarabici* DSM 4947 (13) *Dehalogenimonas lykanthroporepellens* BL-DC-9 (14) *Delftia acidovorans* SPH-1 (15) *Sulfurimonas autotrophica* DSM 16294

*TA* Thermophilic archaea, (1) *Sulfolobus islandicus* L.D.8.5 (2) *Candidatus Caldiarchaeum subterraneum* (3) *Thermofilum pendens Hrk* 5 (4) *Archaeoglobus profundus* DSM 5631 (5) *Ferroglobus placidus* DSM 10642 (6) Aciduliprofundum boonei T469 (7) *Fervidicoccus fontis Kam* 940 (8) *Staphylothermus hellenicus* DSM 12710 (9) *Candidatus Korarchaeum cryptofilum* OPF8 (10) *Thermoproteaceae Vulcanisaeta* (11) *Pyrobaculum arsenaticum* DSM 13514 (12) *Archaeoglobus veneficus* SNP6 (13) *Sulfolobus solfataricus* 98/2 (14) *Sulfolobus islandicus* Y.G.57.14 (15) *Sulfolobus islandicus* M.14.25

** Significant at a level of 1 % of probability (*P* < 0.01)

* Significant at a level of 5 % of probability (0.01 ≤ *P* < 0.05)

*ns* non-significant (*P* ≥ 0.05)

Significant differences were found between the nitrilase/cyanide hydratase sequences of mesophilic bacteria and thermophilic archaea for various physicochemical parameters like number of amino acid residues, molecular weight, positively charged residues (Arg + Lys), aliphatic index and GRAVY. The mesophilic bacterial nitrilase/cyanide hydratase sequences have significant number of amino acid residues as compared to nitrilase/cyanide hydratase sequences of thermophilic archaea (1.09-fold). The molecular weight of nitrilases/cyanide hydratases of mesophilic bacteria was found to be insignificantly high as compared to the thermophilic archaea (1.06-fold). The GRAVY of nitrilase/cyanide hydratase sequences from mesophilic bacteria was found to be significantly high (1.64-fold) as compared to thermophilic archaea. The positively charged residues (Arg + Lys) and aliphatic index values were found to be higher (1.13, 1.16-fold, respectively) in thermophilic archaeal nitrilases/cyanide hydratases as compared to mesophilic bacteria. A statistical analysis shows that the aliphatic index, which is defined as the relative volume of a protein occupied by aliphatic side chains (alanine, valine, isoleucine, and leucine), of proteins of thermophilic bacteria is significantly higher than that of ordinary proteins. The index may be regarded as a positive factor for the increase of thermostability of globular proteins (Atsushi 1980).

Due to diversity of 20 amino acids, and to the incredible number of combinations they offer, proteins differ widely in physicochemical properties as well as in substrate specificity (Sharma et al. 2009). The result of this study has also confirmed that amino acid number and their percent composition in sequences belonging to nitrilase/cyanide hydratase family significantly affect the substrate specificity. Several investigators have focused on the problem of the molecular basis of protein thermostability. A number of physicochemical properties have been attributed to the greater stability of the thermophilic proteins (Jaenicke and Bohm 1998; Ladenstein and Antranikian 1998). These families have an entire spectrum, containing proteins from moderately thermophilic to hyperthermophilic organisms and their mesophilic homologs. Not all the differences observed between the thermophilic and mesophilic proteins are due to thermostability. Results of amino acid analysis of three groups of sequences from nitrilase/cyanide hydrates family are shown in Tables 7, 8 and 9. These enzymes contained all 20 common amino acids. The comparison of the amino acid composition of nitrilases/cyanide hydratases of the mesophilic and thermophilic bacteria has shown that Ala, one of the simplest amino acid, was found to be the predominant residue in mesophilic bacteria and Lys and Phe in thermophilic bacteria. The amino acid Gln (1.4-fold) was observed to be significantly high in thermophilic bacterial nitrilases/cyanide hydratases and the amino acid Val (1.29-fold) was found to be higher in thermophilic archaeal nitrilases/cyanide hydratases. The comparison of the amino acid residues in mesophilic bacteria and thermophilic archaea has also been done and the amino acid Cys is considered to be an important parameter in the calculation of extinction co-efficient of proteins (Sharma et al. 2009) and its content was 1.6 fold higher in mesophilic bacteria as compared to thermophilic archaea. The amino acids Ala, Gln, His and Thr were (1.41, 1.77, 1.44 and 1.29) significantly higher in mesophilic bacteria, while the amino acids, Glu, Leu and Val (1.22, 1.19 and 1.26) were higher in thermophilic archaea.

**Table 7:** Comparison of amino acid composition present in mesophilic and thermophilic bacteria for nitrilase/cyanide hydratase family

Amino acid composition	SS	Microorganisms															(*P* < 0.01)
		1	2	3	4	5	6	7	8	9	10	11	12	13	14	15	
Ala (A)	MB	10.5	10.1	11.5	17.6	8.6	7.9	7.7	10.7	8.6	12.4	15.8	6.5	13.2	7.2	5.8	5.41*
	TB	4.8	7.4	12.8	7.0	4.6	6.4	6.4	6.9	4.5	14.3	12.0	7.9	3.1	6.2	7.8	
Arg (R)	MB	4.5	7.2	10.4	7.9	4.6	4.1	2.6	6.6	4.0	8.0	7.7	5.1	7.8	5.2	2.9	0.02 ns
	TB	6.6	3.0	7.9	5.3	3.1	8.0	6.4	5.2	4.5	6.9	7.8	6.8	5.9	4.3	8.6	
Asn (N)	MB	4.5	4.7	2.6	2.4	3.3	7.1	4.0	3.8	4.3	1.1	2.8	4.3	2.3	4.9	5.8	0.53 ns
	TB	2.6	6.5	3.3	3.7	6.5	4.2	3.0	2.4	5.7	3.7	3.5	4.6	5.5	4.7	3.7	
Asp (D)	MB	4.1	4.3	6.3	6.6	6.3	5.6	5.1	6.4	6.6	5.5	5.6	5.1	5.4	5.2	5.8	3.10 ns
	TB	6.6	4.8	4.5	7.0	5.0	3.4	2.6	6.5	4.9	3.7	3.5	4.3	5.9	5.2	5.6	
Cys (C)	MB	1.1	1.8	0.7	0.3	1.3	2.2	1.1	2.9	1.7	1.8	1.1	2.2	2.3	2.9	1.2	0.052 ns
	TB	0.4	3.9	1.7	0.4	2.3	0.4	1.1	2.1	0.8	1.6	1.6	1.8	2.4	2.3	0.7	
Gln (Q)	MB	3.0	2.9	1.9	2.4	4.6	1.9	4.8	3.8	1.3	6.6	2.5	2.2	1.6	2.6	2.9	1.91 ns
	TB	2.2	1.7	2.1	1.6	3.4	1.9	1.5	4.1	2.4	2.0	1.9	4.3	2.4	2.7	1.9	
Glu (E)	MB	8.6	6.1	9.6	6.9	6.6	6.4	8.1	6.9	5.6	5.8	7.4	8.0	8.6	7.5	7.9	2.89 ns
	TB	10.5	8.7	7.0	7.8	6.9	7.2	9.4	6.2	8.1	6.9	8.9	8.2	6.7	10.5	8.6	
Gly (G)	MB	4.1	8.3	8.5	8.6	7.3	5.2	5.9	7.8	8.6	8.4	9.8	6.5	8.6	9.9	6.2	0.04 ns
	TB	8.7	5.6	7.9	4.9	6.9	7.2	9.7	6.9	7.3	10.6	9.3	6.8	6.3	7.0	6.7	
His (H)	MB	1.1	2.2	2.6	2.4	3.0	1.5	3.3	2.0	2.0	3.6	2.5	1.4	2.3	3.2	2.1	2.07 ns
	TB	3.5	2.2	1.2	2.0	1.5	1.5	1.9	3.4	2.0	2.0	1.9	2.5	0.8	1.9	1.1	
Ile (I)	MB	10.5	5.4	7.4	5.5	7.9	8.6	5.5	6.1	6.0	2.6	5.3	8.3	3.5	6.4	7.5	0.84 ns
	TB	4.4	11.3	4.5	6.1	8.4	8.3	6.4	6.2	11.0	4.9	6.2	5.7	8.2	8.2	7.1	
Leu (L)	MB	8.6	8.3	7.8	9.3	7.0	6.7	9.5	10.1	6.6	13.1	6.0	7.2	8.9	4.6	10.8	1.44 ns
	TB	7.0	9.1	14.0	7.0	10.7	11.4	9.7	7.2	8.1	11.4	8.5	8.9	9.0	7.4	8.6	
Lys (K)	MB	9.4	4.7	2.2	2.8	5.0	6.4	7.0	2.9	6.3	1.1	3.2	6.5	1.9	4.9	7.5	5.14*
	TB	6.6	8.7	4.5	8.6	9.6	7.6	6.4	4.8	10.2	2.4	3.9	4.6	8.2	10.1	7.5	
Met (M)	MB	1.5	1.8	3.3	1.7	3.3	4.1	2.6	1.4	2.7	1.8	2.5	2.5	1.2	4.6	1.7	1.47 ns
	TB	3.1	1.7	1.2	3.3	1.1	1.1	2.2	1.4	2.0	2.4	2.7	1.8	2.0	2.7	2.2	
Phe (F)	MB	3.4	4.0	3.0	3.1	3.3	4.1	3.7	4.0	3.0	1.8	4.2	6.5	3.9	2.9	6.7	8.05**
	TB	4.8	4.8	4.5	9.4	6.9	8.0	7.1	5.2	4.1	4.1	3.9	4.6	7.1	3.9	3.4	
Pro (P)	MB	3.0	5.8	5.2	3.4	5.3	3.0	2.9	6.9	7.6	6.9	3.9	3.3	6.6	5.2	2.5	2.55 ns
	TB	4.4	3.8	5.4	3.7	2.3	3.0	2.6	5.2	3.7	4.5	3.5	5.0	2.4	3.2	6.0	
Ser (S)	MB	7.1	5.1	3.3	4.8	4.6	8.6	8.1	4.6	5.0	4.0	3.9	8.0	3.5	4.6	5.8	0.90 ns
	TB	3.1	5.6	2.5	4.5	4.6	6.8	6.0	3.4	6.1	4.9	3.5	4.6	8.6	4.7	3.4	
Thr (T)	MB	2.6	5.1	3.3	4.8	4.3	5.2	4.8	4.0	5.3	4.4	4.9	3.6	5.4	4.6	3.8	0.02 ns
	TB	4.4	2.4	6.2	6.6	6.1	2.3	3.7	8.9	3.3	3.3	3.1	5.4	3.1	3.5	2.6	
Trp (W)	MB	0.4	0.7	1.5	1.0	0.7	0.7	1.5	1.4	2.0	2.2	1.1	0.7	3.1	1.4	1.7	1.10 ns
	TB	2.2	0.4	0.4	1.6	1.9	0.0	0.4	1.0	0.8	0.4	1.9	0.7	0.8	1.6	1.9	
Tyr (Y)	MB	6.0	3.6	2.2	2.1	5.0	3.0	3.7	2.9	7.0	2.6	1.8	5.4	1.9	5.2	2.9	0.30 ns
	TB	5.7	5.6	2.0	5.7	3.4	5.3	4.5	4.1	4.5	3.7	3.5	2.9	5.5	4.3	3.7	
Val (V)	MB	6.0	7.9	6.7	6.2	7.9	7.5	8.4	4.6	5.6	6.2	8.4	6.5	7.8	6.7	8.3	0.068 ns
	TB	8.7	3.0	5.4	3.7	4.6	6.1	9.0	8.9	6.1	6.1	8.9	8.6	6.3	6.2	10.8	

Substarte specificity: *MB* Mesophilic Bacteria, (1) *Halanaerobium hydrogeniformans* (2) *Desulfotomaculum gibsoniae* DSM 7213 (3) *Thermobacillus composti* KWC4 (4) *Mesorhizobium amorphae* CCNWGS0123 (5) *Serratia sp.* M24T3 (6) *Clostridium papyrosolvens* DSM 2782 (7) *Kangiella koreensis* DSM 16069 (8) *Planctomyces brasiliensis* DSM 5305 (9) *Ethanoligenens harbinense* YUAN-3 (10) *Ferrimonas balearica* DSM 9799 (11) *Sinorhizobium meliloti* AK83 (12) *Flexistipes sinusarabici* DSM 4947 (13) *Dehalogenimonas lykanthroporepellens* BL-DC-9 (14) *Delftia acidovorans* SPH-1 (15) *Sulfurimonas autotrophica* DSM 16294

*TB* Thermophilic Bacteria (1) *Bacillus Smithii* (2) *Caldicellulosiruptor kronotskyensis* 2002 (3) *Ammonifex degensii* KC4 (4) *Kosmotoga olearia* TBF 19.5.1 (5) *Hippea maritima* DSM 10411 (6) *Thermosipho melanesiensis* BI429 (7) *Thermotoga petrophila* RKU-1 (8) *Geobacillus thermoglucosidasius* C56-YS93 (9) *Persephonella marina* EX-H1 (10) *Moorella thermoacetica* ATCC 39073 (11) Thermosinus carboxydivorans Nor1 (12) *Desulfotomaculum kuznetsovii* (13) *Thermodesulfobium narugense* DSM 14796 (14) *Clostridium thermocellum* DSM 2360 (15) *Thermosediminibacter oceani* DSM 16646

** Significant at a level of 1 % of probability (*P* < 0.01)

* Significant at a level of 5 % of probability (0.01 ≤ *P* < 0.05)

*ns* non-significant (*P* ≥ 0.05)

**Table 8:** Comparison of amino acid composition present in thermophilic bacteria and archaea for nitrilase/cyanide hydratase family

Amino acid composition	SS	Microorganisms															(*P* < 0.01)
		1	2	3	4	5	6	7	8	9	10	11	12	13	14	15	
Ala (A)	TA	4.6	8.9	10.8	6.1	7.2	8.2	4.1	5.1	7.5	6.1	12.8	9.6	5.6	6.3	6.3	0.035 ns
	TB	4.8	7.4	12.8	7.0	4.6	6.4	6.4	6.9	4.5	14.3	12.0	7.9	3.1	6.2	7.8	
Arg (R)	TA	5.5	5.6	10.8	5.6	6.4	6.3	5.6	8.1	6.4	8.0	7.4	7.6	5.9	6.7	6.7	1.99 ns
	TB	6.6	3.0	7.9	5.3	3.1	8.0	6.4	5.2	4.5	6.9	7.8	6.8	5.9	4.3	8.6	
Asn (N)	TA	3.8	4.6	1.8	6.1	5.2	4.7	4.4	2.2	3.0	6.8	1.2	2.8	3.0	3.3	3.3	1.19 ns
	TB	2.6	6.5	3.3	3.7	6.5	4.2	3.0	2.4	5.7	3.7	3.5	4.6	5.5	4.7	3.7	
Asp (D)	TA	5.5	3.7	5.0	4.3	3.2	6.3	3.3	7.3	7.2	5.3	3.1	3.2	5.6	5.6	5.9	0.05 ns
	TB	6.6	4.8	4.5	7.0	5.0	3.4	2.6	6.5	4.9	3.7	3.5	4.3	5.9	5.2	5.6	
Cys (C)	TA	0.4	1.1	1.8	1.7	0.8	0.4	0.4	0.4	1.1	1.1	0.8	2.0	1.1	1.1	1.1	3.70 ns
	TB	0.4	3.9	1.7	0.4	2.3	0.4	1.1	2.1	0.8	1.6	1.6	1.8	2.4	2.3	0.7	
Gln (Q)	TA	1.7	4.4	0.4	1.3	2.0	2.4	1.5	2.2	1.1	0.8	1.9	2.0	1.9	1.1	1.1	4.37*
	TB	2.2	1.7	2.1	1.6	3.4	1.9	1.5	4.1	2.4	2.0	1.9	4.3	2.4	2.7	1.9	
Glu (E)	TA	8.4	7.8	11.5	7.4	11.6	7.5	9.6	6.2	8.3	5.7	9.7	10.8	9.3	9.6	9.6	1.77 ns
	TB	10.5	8.7	7.0	7.8	6.9	7.2	9.4	6.2	8.1	6.9	8.9	8.2	6.7	10.5	8.6	
Gly (G)	TA	8.0	7.4	8.6	6.5	6.8	5.5	5.9	5.1	8.3	9.5	9.7	7.2	5.6	5.6	5.6	0.60 ns
	TB	8.7	5.6	7.9	4.9	6.9	7.2	9.7	6.9	7.3	10.6	9.3	6.8	6.3	7.0	6.7	
His (H)	TA	0.4	3.7	2.5	2.2	0.8	0.8	0.4	2.6	1.9	0.8	1.6	1.2	1.9	1.9	1.9	1.58 ns
	TB	3.5	2.2	1.2	2.0	1.5	1.5	1.9	3.4	2.0	2.0	1.9	2.5	0.8	1.9	1.1	
Ile (I)	TA	10.9	7.8	3.6	8.2	7.6	8.6	9.6	9.9	6.4	9.1	1.9	6.4	8.9	8.5	8.5	0.53 ns
	TB	4.4	11.3	4.5	6.1	8.4	8.3	6.4	6.2	11.0	4.9	6.2	5.7	8.2	8.2	7.1	
Leu (L)	TA	10.9	9.6	10.0	10.4	10.8	7.8	11.1	8.4	9.4	9.8	10.9	10.0	10.0	9.6	9.6	1.50 ns
	TB	7.0	9.1	14.0	7.0	10.7	11.4	9.7	7.2	8.1	11.4	8.5	8.9	9.0	7.4	8.6	
Lys (K)	TA	8.0	4.8	1.8	10.4	6.4	6.7	8.1	5.9	6.0	1.9	5.0	5.2	7.8	7.0	7.0	0.58 ns
	TB	6.6	8.7	4.5	8.6	9.6	7.6	6.4	4.8	10.2	2.4	3.9	4.6	8.2	10.1	7.5	
Met (M)	TA	1.3	3.0	1.1	1.7	2.8	2.7	1.9	2.6	2.4	1.9	1.2	1.6	1.5	1.9	1.9	0.15 ns
	TB	3.1	1.7	1.2	3.3	1.1	1.1	2.2	1.4	2.0	2.4	2.7	1.8	2.0	2.7	2.2	
Phe (F)	TA	4.2	42.6	3.2	5.2	4.4	8.2	2.6	4.4	6.4	2.7	4.7	4.0	5.2	5.2	5.2	2.92 ns
	TB	4.8	4.8	4.5	9.4	6.9	8.0	7.1	5.2	4.1	4.1	3.9	4.6	7.1	3.9	3.4	
Pro (P)	TA	5.9	4.8	3.2	4.8	4.0	4.3	4.1	4.4	5.3	2.7	5.8	4.4	3.3	3.3	3.3	0.97 ns
	TB	4.4	3.8	5.4	3.7	2.3	3.0	2.6	5.2	3.7	4.5	3.5	5.0	2.4	3.2	6.0	
Ser (S)	TA	3.4	4.4	6.1	3.0	4.0	3.1	9.6	6.6	6.4	6.8	3.1	3.2	6.7	4.6	5.6	0.29 ns
	TB	3.1	5.6	2.5	4.5	4.6	6.8	6.0	3.4	6.1	4.9	3.5	4.6	8.6	4.7	3.4	
Thr (T)	TA	2.1	4.1	0.7	2.2	1.6	3.5	3.3	4.0	2.6	5.7	3.9	4.8	3.7	4.1	4.1	2.63 ns
	TB	4.4	2.4	6.2	6.6	6.1	2.3	3.7	8.9	3.3	3.3	3.1	5.4	3.1	3.5	2.6	
Trp (W)	TA	0.8	1.0	1.4	0.4	0.4	0.8	2.2	1.1	0.4	1.5	1.2	0.8	1.5	1.6	1.5	0.18 ns
	TB	2.2	0.4	0.4	1.6	1.9	0.0	0.4	1.0	0.8	0.4	1.9	0.7	0.8	1.6	1.9	
Tyr (Y)	TA	3.8	1.4	3.9	3.0	3.2	4.3	3.7	6.6	2.6	5.5	3.5	2.8	5.6	5.6	5.2	0.21 ns
	TB	5.7	5.6	2.0	5.7	3.4	5.3	4.5	4.1	4.5	3.7	3.5	2.9	5.5	4.3	3.7	
Val (V)	TA	10.5	8.5	11.8	9.5	10.8	7.8	8.5	7.0	7.2	9.5	10.9	10.0	6.3	6.7	6.7	7.01*
	TB	8.7	3.0	5.4	3.7	4.6	6.1	9.0	8.9	6.1	6.1	8.9	8.6	6.3	6.2	10.8	

Substarte specificity: *TA* Thermophilic archaea, (1) *Sulfolobus islandicus* L.D.8.5 (2) *Candidatus Caldiarchaeum subterraneum* (3) *Thermofilum pendens Hrk* 5 (4) *Archaeoglobus profundus* DSM 5631 (5) *Ferroglobus placidus* DSM 10642 (6) *Aciduliprofundum boonei* T469 (7) *Fervidicoccus fontis Kam* 940 (8) *Staphylothermus hellenicus* DSM 12710 (9) *Candidatus Korarchaeum cryptofilum* OPF8 (10) *Thermoproteaceae Vulcanisaeta* (11) *Pyrobaculum arsenaticum* DSM 13514 (12) *Archaeoglobus veneficus* SNP6 (13) *Sulfolobus solfataricus* 98/2 (14) *Sulfolobus islandicus* Y.G.57.14 (15) Sulfolobus islandicus M.14.25

*TB* Thermophilic bacteria (1) *Bacillus Smithii* (2) *Caldicellulosiruptor kronotskyensis* 2002 (3) *Ammonifex degensii* KC4 (4) *Kosmotoga olearia* TBF 19.5.1 (5) *Hippea maritima* DSM 10411 (6) *Thermosipho melanesiensis* BI429 (7) *Thermotoga petrophila* RKU-1 (8) *Geobacillus thermoglucosidasius* C56-YS93 (9) *Persephonella marina* EX-H1 (10) *Moorella thermoacetica* ATCC 39073 (11) *Thermosinus carboxydivorans* Nor1 (12) *Desulfotomaculum kuznetsovii* (13) *Thermodesulfobium narugense* DSM 14796 (14) *Clostridium thermocellum* DSM 2360 (15) *Thermosediminibacter oceani* DSM 16646

** Significant at a level of 1 % of probability (*P* < 0.01)

* Significant at a level of 5 % of probability (0.01 ≤ *P* < 0.05)

*ns* non-significant (*P* ≥ 0.05)

**Table 9:** Comparison of amino acid composition present in mesophilic bacteria and thermophilic archaea for nitrilase/cyanide hydratase family

Amino acid composition	SS	Microorganisms															(*P* < 0.01)
		1	2	3	4	5	6	7	8	9	10	11	12	13	14	15	
Ala (A)	MB	10.5	10.1	11.5	17.6	8.6	7.9	7.7	10.7	8.6	12.4	15.8	6.5	13.2	7.2	5.8	7.54*
	TA	4.6	8.9	10.8	6.1	7.2	8.2	4.1	5.1	7.5	6.1	12.8	9.6	5.6	6.3	6.3	
Arg (R)	MB	4.5	7.2	10.4	7.9	4.6	4.1	2.6	6.6	4.0	8.0	7.7	5.1	7.8	5.2	2.9	1.89 ns
	TA	5.5	5.6	10.8	5.6	6.4	6.3	5.6	8.1	6.4	8.0	7.4	7.6	5.9	6.7	6.7	
Asn (N)	MB	4.5	4.7	2.6	2.4	3.3	7.1	4.0	3.8	4.3	1.1	2.8	4.3	2.3	4.9	5.8	0.05 ns
	TA	3.8	4.4	1.8	6.1	5.2	4.7	4.4	2.2	3.0	6.8	1.2	2.8	3.0	3.3	3.3	
Asp (D)	MB	4.1	4.3	6.3	6.6	6.3	5.6	5.1	6.4	6.6	5.5	5.6	5.1	5.4	5.2	5.8	2.22 ns
	TA	5.5	3.7	5.0	4.3	3.2	6.3	3.3	7.3	7.2	5.3	3.1	3.2	5.6	5.6	5.9	
Cys (C)	MB	1.1	1.8	0.7	0.3	1.3	2.2	1.1	2.9	1.7	1.8	1.1	2.2	2.3	2.9	1.2	6.80*
	TA	0.4	1.1	1.8	1.7	0.8	0.4	0.4	0.4	1.1	1.1	0.8	2.0	1.1	1.1	1.1	
Gln (Q)	MB	3.0	2.9	1.9	2.4	4.6	1.9	4.8	3.8	1.3	6.6	2.5	2.2	1.6	2.6	2.9	8.56**
	TA	1.7	4.4	0.4	1.3	2.0	2.4	1.5	2.2	1.1	0.8	1.9	2.0	1.9	1.1	1.1	
Glu (E)	MB	8.6	6.1	9.6	6.9	6.6	6.4	8.1	6.9	5.6	5.8	7.4	8.0	8.6	7.5	7.9	7.98**
	TA	8.4	7.8	11.5	7.4	11.6	7.5	9.6	6.2	0.3	5.7	9.7	10.8	9.3	9.6	9.6	
Gly (G)	MB	4.1	8.3	8.5	8.6	7.3	5.2	5.9	7.8	8.6	8.4	9.8	6.5	8.6	9.9	6.2	0.92 ns
	TA	8.0	7.4	8.6	6.5	6.8	5.5	5.9	5.1	8.3	9.5	9.7	7.2	5.6	5.6	5.6	
His (H)	MB	1.1	2.2	2.6	2.4	3.0	1.5	3.3	2.0	2.0	3.6	2.5	1.4	2.3	3.2	2.1	5.49*
	TA	0.4	3.7	2.5	2.2	0.8	0.8	0.4	2.6	1.9	0.8	1.6	1.2	1.9	1.9	1.9	
Ile (I)	MB	10.5	5.4	7.4	5.5	7.9	8.6	5.5	6.1	6.0	2.6	5.3	8.3	3.5	6.4	7.5	2.60 ns
	TA	10.9	7.8	3.6	8.2	7.6	8.6	9.6	9.9	6.4	9.1	1.9	6.4	8.9	8.5	8.5	
Leu (L)	MB	8.6	8.3	7.8	9.3	7.0	6.7	9.5	10.1	6.6	13.1	6.0	7.2	8.9	4.6	10.8	7.07*
	TA	10.9	9.6	10.0	10.4	10.8	7.8	11.1	8.4	9.4	9.8	10.9	10.0	10.0	9.6	9.6	
Lys (K)	MB	9.4	4.7	2.2	2.8	5.0	6.4	7.0	2.9	6.3	1.1	3.2	6.5	1.9	4.9	7.5	2.52 ns
	TA	8.0	4.8	1.8	10.4	6.4	6.7	8.1	5.9	6.0	1.9	5.0	5.2	7.8	7.0	7.0	
Met (M)	MB	1.5	1.8	3.3	1.7	3.3	4.1	2.6	1.4	2.7	1.8	2.5	2.5	1.2	4.6	1.7	2.35 ns
	TA	1.3	3.0	1.1	1.7	2.8	2.7	1.9	2.6	2.4	1.9	1.2	1.8	1.5	1.9	1.9	
Phe (F)	MB	3.4	4.0	3.0	3.1	3.3	4.1	3.7	4.0	3.0	1.8	4.2	6.5	3.9	2.9	6.7	1.91 ns
	TA	4.2	2.6	3.2	5.2	4.4	8.2	2.6	4.4	6.4	2.7	4.7	4.0	5.2	5.2	5.2	
Pro (P)	MB	3.0	5.8	5.2	3.4	5.3	3.0	2.9	6.9	7.6	6.9	3.9	3.3	6.6	5.2	2.5	1.06 ns
	TA	5.9	4.8	3.2	4.8	4.0	4.3	4.1	4.4	5.3	2.7	5.8	4.4	3.3	3.3	3.3	
Ser (S)	MB	7.1	5.1	3.3	4.8	4.6	8.6	8.1	4.6	5.0	4.0	3.9	8.0	3.5	4.6	5.8	0.11 ns
	TA	3.4	4.4	6.1	3.0	4.0	3.1	9.6	6.6	6.4	6.8	3.1	3.2	6.7	5.6	5.6	
Thr (T)	MB	2.6	5.1	3.3	4.8	4.3	5.2	4.8	4.0	5.3	4.4	4.9	3.6	5.4	4.6	3.8	6.99*
	TA	2.1	4.1	0.7	2.2	1.6	3.5	3.3	4.0	2.6	5.7	3.9	4.8	3.7	4.1	4.1	
Trp (W)	MB	0.4	0.7	1.5	1.0	0.7	0.7	1.5	1.4	2.0	2.2	1.1	0.7	3.1	1.4	1.7	0.40 ns
	TA	0.8	1.9	1.4	0.4	0.4	0.8	2.2	1.1	0.8	1.5	1.2	0.8	1.5	1.6	1.5	
Tyr (Y)	MB	6.0	3.6	2.2	2.1	5.0	3.0	3.7	2.9	7.0	2.6	1.8	5.4	1.9	5.2	2.9	0.41 ns
	TA	3.8	1.4	3.9	3.0	3.2	4.3	3.7	6.6	2.6	5.5	3.5	2.8	5.6	5.6	5.2	
Val (V)	MB	6.0	7.9	6.7	6.2	7.9	7.5	8.4	4.6	5.6	6.2	8.4	6.5	7.8	6.7	8.3	10.74**
	TA	10.5	8.5	11.8	9.5	10.8	7.8	8.5	7.0	7.2	9.5	10.9	10.0	6.3	6.7	6.7	

Substarte specificity: *MB* Mesophilic Bacteria, (1) *Halanaerobium hydrogeniformans* (2) *Desulfotomaculum gibsoniae* DSM 7213 (3) *Thermobacillus composti* KWC4 (4) *Mesorhizobium amorphae* CCNWGS0123 (5) *Serratia* sp. M24T3 (6) *Clostridium papyrosolvens* DSM 2782 (7) *Kangiella koreensis* DSM 16069 (8) *Planctomyces brasiliensis* DSM 5305 (9) *Ethanoligenens harbinense* YUAN-3 (10) *Ferrimonas balearica* DSM 9799 (11) *Sinorhizobium meliloti* AK83 (12) *Flexistipes sinusarabici* DSM 4947 (13) *Dehalogenimonas lykanthroporepellens* BL-DC-9 (14) *Delftia acidovorans* SPH-1 (15) *Sulfurimonas autotrophica* DSM 16294

*TA* Thermophilic archaea, (1) *Sulfolobus islandicus* L.D.8.5 (2) *Candidatus Caldiarchaeum subterraneum* (3) *Thermofilum pendens* Hrk 5 (4) *Archaeoglobus profundus* DSM 5631 (5) *Ferroglobus placidus* DSM 10642 (6) *Aciduliprofundum boonei* T469 (7) *Fervidicoccus fontis* Kam 940 (8) *Staphylothermus hellenicus* DSM 12710 (9) *Candidatus Korarchaeum cryptofilum* OPF8 (10) *Thermoproteaceae Vulcanisaeta* (11) *Pyrobaculumarsenaticum* DSM 13514 (12) *Archaeoglobus veneficus* SNP6 (13) *Sulfolobus solfataricus* 98/2 (14) *Sulfolobus islandicus Y.G.57.14* (15) *Sulfolobus islandicus* M.14.25

* Significant at a level of 1 % of probability (*P* < 0.01)

* Significant at a level of 5 % of probability (0.01 ≤ *P* < 0.05)

*ns* non-significant (*P* ≥ 0.05)

Analysis of the amino acid composition of helices in thermophilic proteins appears to indicate that a number of Gly residues are enhanced as compared to those of mesophilic proteins (Warren and Petsko 1995). Some workers found that the decreased Gln content may minimize deamidation which results in increased thermostability of proteins. It has also been suggested that Lys → Arg and Ser → Ala are the most frequent mutations in mesophilic to thermophilic substitutions (Arias and Argos 1989). Ala is the best helix-forming residue (Kumar and Bansal 1998; Best et al. 2012), however, the decreased Ala content in thermophilic proteins is still unknown. The most significant observation in the present analysis was that the number of Glu and Lys residues was increased in thermophiles in comparison with mesophiles. The juxtaposition of these residues is perhaps important in imparting thermal stability (Parthasarathy and Murthy 2000). These residues may be appropriate candidates for site-specific mutations leading to enhanced stability.

## Conclusion

A number of physicochemical properties of amino acid sequences belonging to nitrilase/cyanide hydratase family from mesophiles and thermophiles have been deduced. They mainly differ in the total number of amino acid, molecular weight, pI, negatively and positively charged residues, aliphatic index, GRAVY and composition of amino acids. The presence of Ala, Gln, His and Thr in mesophilic organisms and the amino acids, Glu, Leu and Val in thermophilic organisms clearly indicate them to be in mesophiles and thermophiles, respectively. As discriminating thermophilic proteins from their mesophilic counterparts is a challenging task, the results of the present work will be quite useful in prediction and selection of the nitrilase/cyanide hydratases for further basic and applied research and it would also help in designing stable proteins.
